# Prosthetic Options for Full-Mouth Implant Rehabilitation: A Contemporary Review

**DOI:** 10.7759/cureus.99222

**Published:** 2025-12-14

**Authors:** Deeksha Surabathula, Sanket Birajdar, Mridula Joshi, Mirella Vaz, Nilesha Kadam

**Affiliations:** 1 Prosthodontics and Crown and Bridge, Bharati Vidyapeeth (Deemed to be Universty) Dental College and Hospital, Navi Mumbai, IND

**Keywords:** all-on-four concept, all-on-six concept, full-mouth rehabilitation, implant-supported overdenture, removable full-arch prosthesis

## Abstract

Full-mouth implant rehabilitation offers a transformative solution for edentulous patients, restoring function, esthetics, and quality of life. With evolving materials and digital technologies, clinicians today can choose from a spectrum of fixed and removable implant-supported prostheses tailored to individual clinical conditions. This review evaluates prosthetic designs, including All-on-Four and All-on-Six concepts, fixed monolithic zirconia and metal-supported prostheses, implant-supported overdentures, and retention mechanisms such as bar, stud, and locator attachments. Emphasis is placed on biomechanical considerations, material selection, digital workflows, and long-term clinical outcomes.

## Introduction and background

Edentulism continues to be a major global oral health burden, affecting mastication, speech, esthetics, nutrition, and overall quality of life. In addition to functional impairment, total loss of natural dentition causes psychological discomfort, social disengagement, and low self-esteem. Although conventional complete dentures have long served as the primary treatment modality for edentulous patients, their limitations, such as compromised stability, insufficient retention, accelerated ridge resorption, and reduced patient satisfaction, are well documented. The paradigm of managing edentulism has evolved substantially with the advent of osseointegrated implants, which offer superior biomechanical support, functional efficiency, and long-term predictability.

Over the past two decades, full-mouth implant rehabilitation has transformed into a highly predictable and evidence-based treatment approach, largely due to advancements in implant surface technology, surgical protocols, and prosthetic materials. The precision and effectiveness of full-arch restorations have been further improved by concurrent advancements in computer-assisted planning, digital impressions, guided surgery, and computer-aided design-computer-aided manufacturing (CAD-CAM) fabrication. Today, clinicians can choose from a wide range of prosthetic solutions, including fixed implant-supported bridges, metal-acrylic hybrid prostheses, monolithic zirconia frameworks, bar-retained overdentures, and attachment-based removable designs, each with distinct biomechanical, esthetic, and maintenance considerations.

Given the diversity of available options and the rapid evolution of materials and techniques, an updated review of prosthetic strategies is essential for evidence-based decision-making. This article aims to analyze current prosthetic options for full-mouth implant-supported rehabilitation, with emphasis on clinical indications, biomechanics, esthetic outcomes, long-term survival, complications, and patient-reported satisfaction. Understanding these parameters enables clinicians to select the most appropriate and predictable treatment modality tailored to the anatomical, functional, and psychological needs of each edentulous patient.

## Review

Prosthetic options in full-mouth implants

Prosthetic options in full-mouth implant rehabilitation encompass a wide range of fixed and removable designs, selected based on anatomical limitations, esthetic demands, patient preferences, and functional goals. Fixed implant-supported prostheses, such as full-arch screw-retained restorations, are frequently delivered using protocols such as the All-on-Four [1] or All-on-Six [2] concepts, which offer high success rates with reported implant survival ranging from 92% to 99% over 5-10 years, conducted in a prospective study, and prosthetic survival around 95% conducted by Elysad et al. in an in-vitro study [3]. These are commonly fabricated using high-strength materials such as monolithic zirconia or cobalt-chromium frameworks veneered with ceramic or acrylic [4].

In addition to conventional All-on-Four and All-on-Six designs, full-arch rehabilitation in cases of severe maxillary atrophy often requires the use of specialized implant modalities such as zygomatic and pterygoid implants. These implants expand treatment possibilities when posterior maxillary bone is inadequate and allow delivery of fixed FP3-type prostheses without grafting.

Alternatively, removable implant-supported prostheses, including implant-retained overdentures, provide a cost-effective and hygienic solution for patients, especially those with systemic health concerns or limited dexterity. These can be supported by two to six implants using various attachment systems, such as studs, bars, or locators [5,6], with implant and prosthesis survival rates also ranging between 90% and 96% and approximately 95%, respectively, as done in a longitudinal study, with a follow-up period of 10 years [7,8]. For patients presenting with severely resorbed ridges, treatment options include the use of zygomatic implants, tilted implants, or bone augmentation techniques (e.g., guided bone regeneration, sinus lift procedures) to enhance bone volume and support fixed prosthetic rehabilitation. In such cases, removable prostheses may also be preferred when surgical interventions are contraindicated. Ultimately, the prosthetic selection must be tailored through an evidence-based, multidisciplinary approach that balances anatomical feasibility, biomechanical requirements, long-term maintenance, and patient satisfaction.

Fixed full-arch prosthetic options

One of the most comprehensive systems for classifying implant prostheses was proposed by Carl Misch, which includes both fixed prostheses (FP1, FP2, FP3) and removable prostheses (RP4, RP5). FP1 replaces only the crown portion of missing teeth and is used when bone and gingival tissues are preserved. FP2 replaces the crowns and part of the root structure and is indicated in moderate ridge resorption. FP3 prostheses replace both teeth and soft tissues and are widely used in full-arch implant cases, especially in severely resorbed ridges where pink porcelain or acrylic is used to replicate lost gingiva. RP4 refers to a removable prosthesis that is fully implant-supported and is used in bar overdenture designs. RP5 prostheses are implant-mucosa-supported and use attachments such as locators, offering good retention with fewer implants [3].

Among the available treatment options, the most frequently used prosthetic designs for full-mouth implant rehabilitation include FP3-type fixed prostheses, such as the All-on-Four or All-on-Six configurations [2]. These involve the strategic placement of four to six implants per arch to support a full-arch prosthesis. Hybrid prostheses using acrylic or zirconia are typically screw-retained and help in restoring both esthetics and function [4]. In cases with anatomical or financial limitations, removable prostheses such as bar-retained overdentures (RP4) or locator-retained overdentures (RP5) provide viable alternatives that are simpler to fabricate and maintain [5,7].

All-on-Four and All-on-Six Concepts

**Figure 1:**
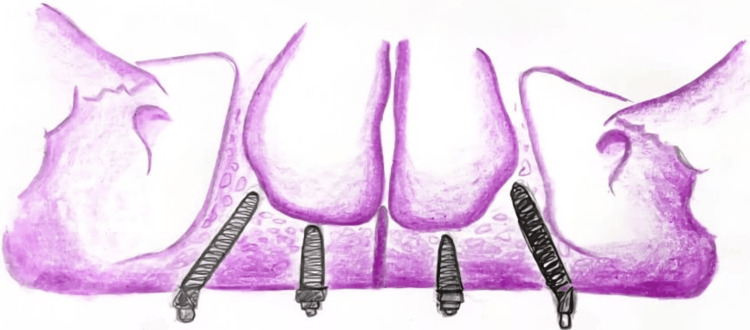
Illustration with all on four concept maxilla Image credits: Deeksha Surabathula. Illustrated using the Wikimedia Commons website

**Figure 2:**
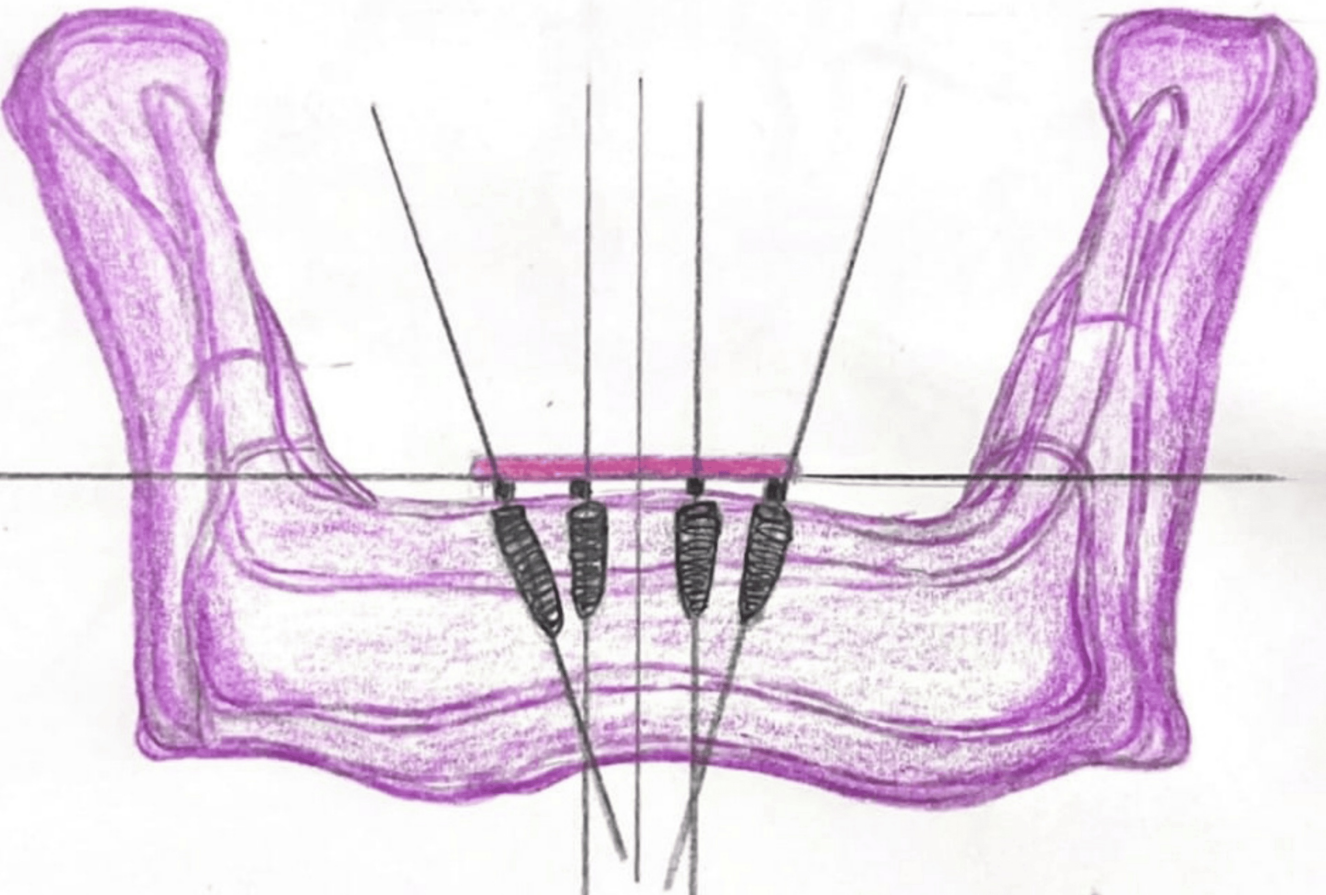
Illustration with all on four concept mandible Image Credits: Deeksha Surabathula. Illustrated using Ref [10]

The All-on-Four concept, introduced and popularized by Maló et al. [9], involves the strategic placement of four dental implants to support a full-arch prosthesis - two implants placed vertically in the anterior region and two posterior implants inserted at an angle (typically 30°-45°) to avoid anatomical structures such as the maxillary sinus or inferior alveolar nerve [1], as shown in Figures 1-2. This angulation increases the anteroposterior spread, allowing for better support of distal cantilevers and reducing the need for extensive bone grafting. The approach is well-suited for patients with moderate bone atrophy and enables immediate loading, which significantly reduces treatment time and improves early prosthetic function.

A defining feature of the All-on-Four protocol is its suitability for immediate loading. Unlike conventional protocols that require a healing period of three to six months before prosthesis placement, this approach allows for the delivery of a provisional, screw-retained fixed prosthesis within 24-48 hours following surgery [6]. This immediate restoration not only improves patient comfort, esthetics, and function from the outset but also reduces the total treatment time significantly. Maló et al. reported high implant survival rates (over 95%) even with immediate loading, both in the maxilla and mandible, with follow-ups extending to 10 years, demonstrating the predictability and long-term success of the approach.

**Figure 3:**
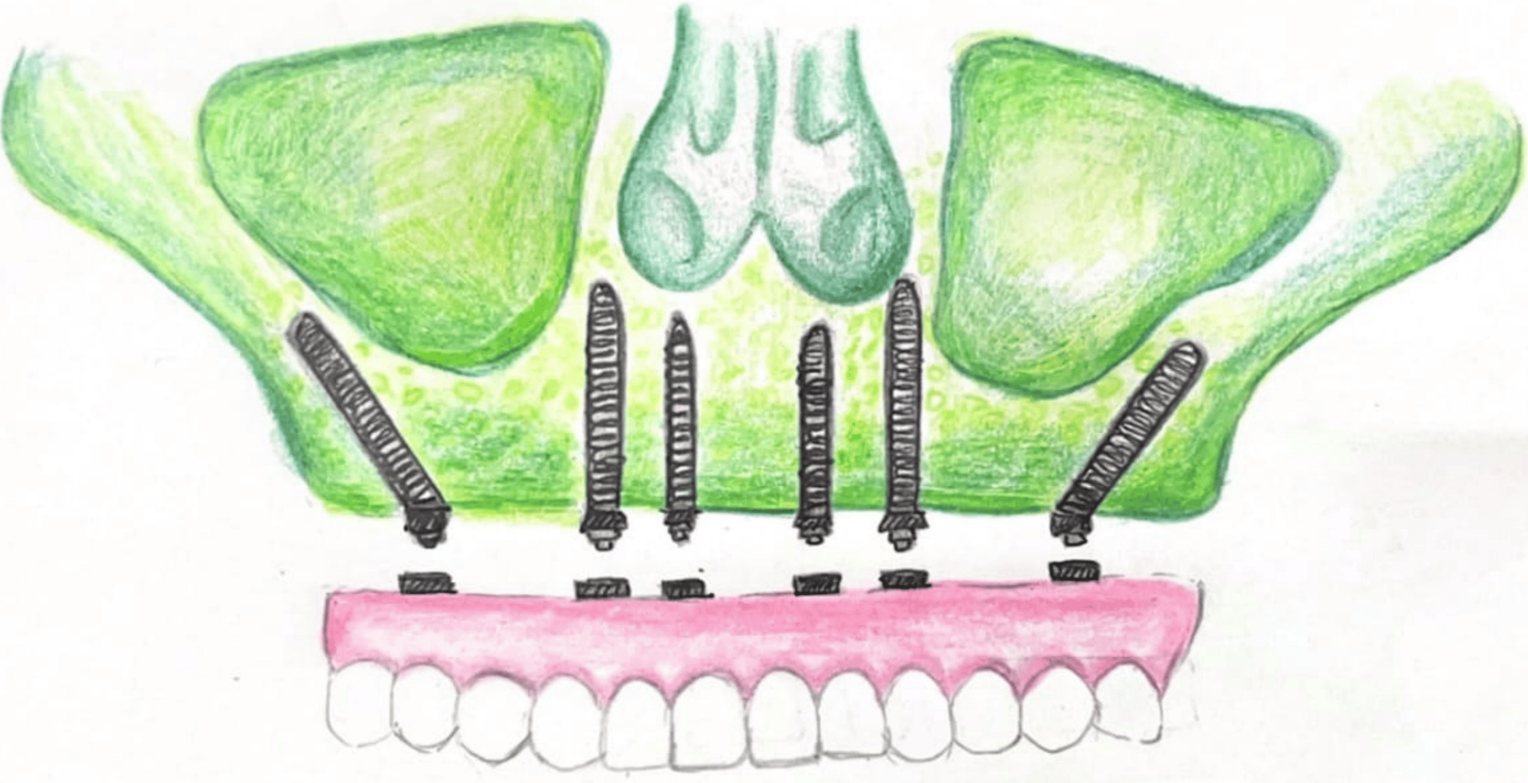
Illustration with all on six concept maxilla Image credits: Deeksha Surabathula. Illustrated using the Wikimedia Commons website

**Figure 4:**
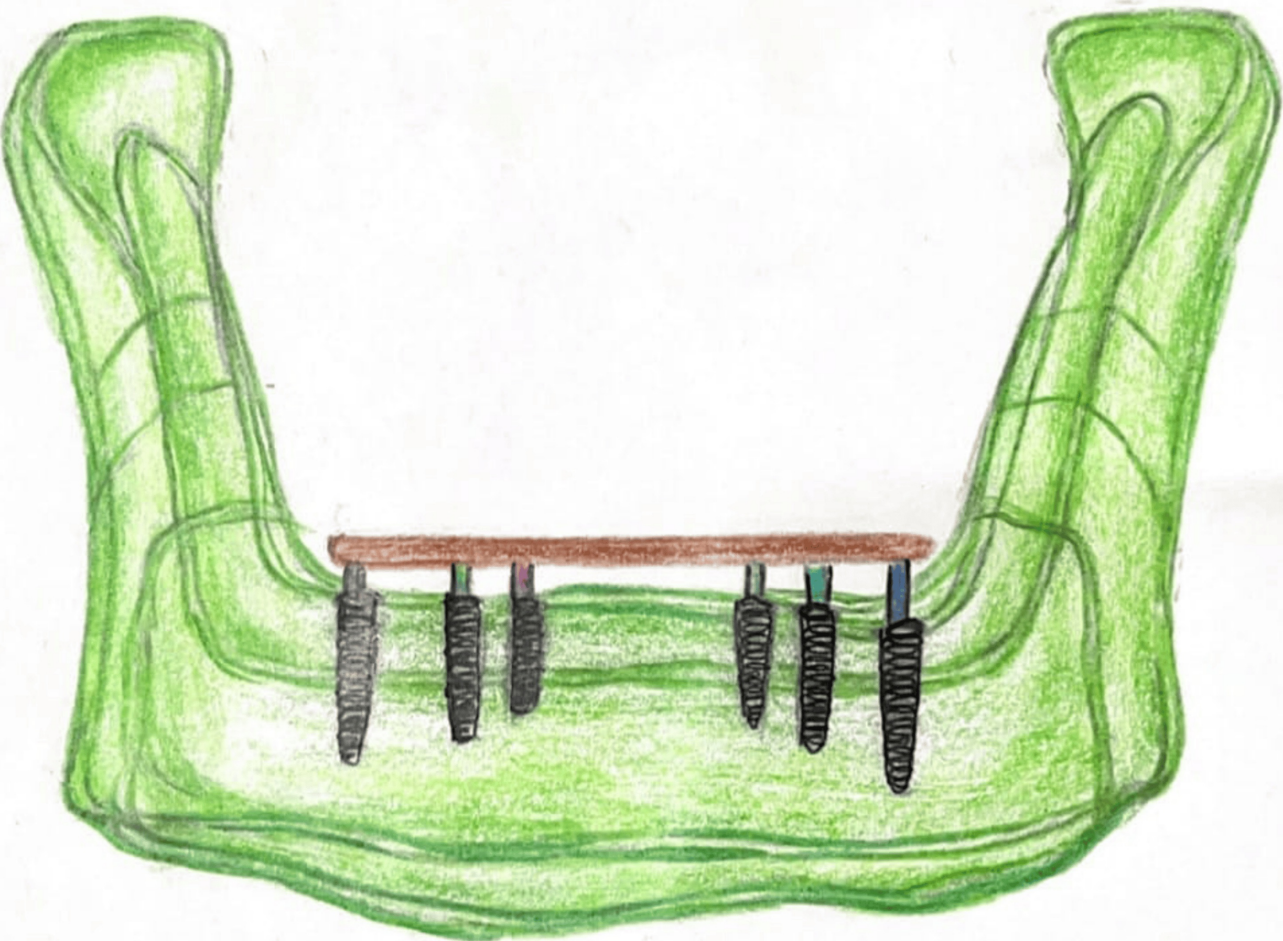
Illustration with all on six concept mandible Image credits: Deeksha Surabathula. Illustrated using Ref [10]

In contrast, the All-on-Six concept utilizes six axially placed implants, offering increased surface area for load distribution, as shown in Figures 3-4. The additional implants enhance biomechanical stability, especially in high-load situations such as bruxism, large occlusal schemes, or dense cortical bone regions [2]. Finite element analysis (FEA) studies have shown that All-on-Six configurations produce more uniform stress distribution across the prosthesis and peri-implant bone compared to All-on-Four, thereby reducing the risk of implant micromotion, screw loosening, or prosthesis fracture [2,10], as presented in Table 1.

Zygomatic and pterygoid implants in full-mouth prosthetic options

Zygomatic Implants

Zygomatic implants are indicated in cases of severe maxillary atrophy where posterior alveolar bone is insufficient for conventional implant placement. These long implants (30-52 mm) engage the dense cortical bone of the zygoma, providing exceptional primary stability [11,12]. They eliminate the need for sinus grafting and permit immediate loading of FP3-type full-arch prostheses. Prosthetically, emergence profiles must be carefully designed to avoid bulky contours and ensure adequate hygiene access. Quad-zygoma configurations enhance cross‑arch stabilization and provide robust support for full-arch zirconia or titanium frameworks.

Pterygoid Implants

Pterygoid implants traverse the maxillary tuberosity and anchor into the pterygoid process of the sphenoid bone. Their placement avoids sinus augmentation, restores posterior support, and significantly reduces distal cantilevers. They improve the anteroposterior spread of implants and reduce biomechanical stress on full-arch prostheses [11,12]. In FP3 prostheses, pterygoid implants enhance occlusal stability, lengthen the functional occlusal table, and improve masticatory efficiency.

Both implant types greatly broaden treatment options in atrophic maxillae and integrate seamlessly with modern full-arch frameworks, including monolithic zirconia, titanium‑zirconia hybrids, and high‑performance polymers, providing strong posterior support and improved long‑term prosthetic outcomes.

Monolithic zirconia and metal-ceramic prostheses: Monolithic zirconia prostheses are milled from a single block of zirconia, ensuring superior strength and reduced chipping compared to layered zirconia [4]. They offer excellent esthetics and biocompatibility but may be prone to wear on opposing dentition if not properly polished. Ceramic prostheses, particularly those fabricated with cobalt-chromium frameworks, allow for veneering porcelain and better color matching [10]. Hybrid prostheses incorporating a metal framework and acrylic or composite resin teeth are cost-effective and easier to repair [1,2,6,7].

Screw-retained vs. cement-retained prostheses: Screw-retained prostheses allow retrievability, which is advantageous for maintenance and managing peri-implant complications. Cement-retained prostheses, on the other hand, offer better esthetics due to the absence of screw-access holes but are associated with risks of excess cement leading to peri-implantitis. Proper venting, cementation techniques, and use of retrievable cements are essential when choosing cement retention [13]. Table 1 presents a comparison of overdenture attachment systems

**Table 1:** Comparison of overdenture attachment systems The table has been created based on the information provided in Refs [14,15].

Feature [14,15]	Locator Attachment [14,15]	Bar-Clip Attachment [14,15]	Ball Attachment [14,15]
Retention	High	Very High	Moderate
Hygiene Maintenance	Easier	Challenging	Moderate
Space Requirement	Minimal	More vertical space needed	Minimal
Cost	Moderate	Higher	Low
Repair/Replacement	Easier	Requires lab intervention	Moderate

Removable full-arch prosthetic options

Implant-Supported Overdentures

Implant-supported overdentures are indicated for patients seeking improved retention and stability without committing to fixed restorations. Typically supported by two to four implants, overdentures improve masticatory efficiency, phonetics, and patient satisfaction. The selection of attachment type significantly affects maintenance and long-term success [14,15].

Bar, Stud, and Locator Attachments

Bar attachments splint implants together and provide strong retention but require greater interarch space and can complicate hygiene [3,14]. Stud attachments, such as ball or locator systems, are simple and allow for individual implant function, as shown in Table 2. Locator attachments are self-aligning and popular due to their low profile and ease of use [3,5]. Magnetic attachments, although less common, are an option for patients with dexterity issues [14].

**Table 2:** Properties of common framework materials The table has been created based on the information provided in Ref [8].

Material [8]	Strength [8]	Esthetics [8]	Flexural Resistance [8]	Cost [8]	Long-Term Data [8]	Preferred Use Case [8]
Zirconia	High	Excellent	Brittle if thin	High	Extensive	High esthetic demand, anterior region, full-arch monolithic restorations
Cobalt–Chromium	Very High	Moderate	Excellent	Moderate	Extensive	Full-arch frameworks, posterior regions, hybrid prostheses requiring rigidity
PEEK	Moderate	Good	High flexibility	Moderate	Limited	Patients with bruxism, TMJ disorders, or metal allergies; metal-free framework option
BioHPP (PEEK reinforced with ceramic fillers)	Moderate–High	Good	High flexibility	Moderate–High	Limited–Moderate	Metal-free frameworks with improved wear resistance and polishability; suitable for allergic patients
Acrylic Resin	Low–Moderate	Good	Low	Low	Extensive (in denture use, limited in implant frameworks)	Interim prostheses, low-cost solutions, cases requiring easy modification or repair

Material

The selection of prosthetic material should consider strength, esthetics, cost, and wear characteristics. Zirconia, polyetheretherketone (PEEK), cobalt-chromium, titanium, and hybrid composites are commonly used. Each material has its own indications depending on functional load, esthetic zone, and patient parafunctional habits [8], as presented in Table 3.

**Table 3:** Properties of common framework materials The table has been created based on the information provided in Reg [8]. PEEK: polyetheretherketone

Material [8]	Strength [8]	Esthetics [8]	Flexural Resistance [8]	Cost [8]	Long-Term Data [8]	Preferred Use Case [8]
Zirconia	High	Excellent	Brittle if thin	High	Extensive	High esthetic demand, anterior region, full-arch monolithic restorations
Cobalt–Chromium	Very High	Moderate	Excellent	Moderate	Extensive	Full-arch frameworks, posterior regions, hybrid prostheses requiring rigidity
PEEK	Moderate	Good	High flexibility	Moderate	Limited	Patients with bruxism, TMJ disorders, or metal allergies; metal-free framework option
BioHPP (PEEK reinforced with ceramic fillers)	Moderate–High	Good	High flexibility	Moderate–High	Limited–Moderate	Metal-free frameworks with improved wear resistance and polishability; suitable for allergic patients
Acrylic Resin	Low–Moderate	Good	Low	Low	Extensive (in denture use, limited in implant frameworks)	Interim prostheses, low-cost solutions, cases requiring easy modification or repair

Prosthetic Protocol in Full-Mouth Implants

The prosthetic protocol in full-mouth implant rehabilitation follows a systematic and sequential approach to ensure functional and esthetic success. It begins with a thorough pre-treatment evaluation involving detailed clinical examination, radiographic analysis (e.g., CBCT), diagnostic impressions or intraoral scans, and a diagnostic wax-up to aid in prosthetically driven treatment planning. In many cases, a surgical guide is fabricated to assist in accurate implant placement. Following this, implants are placed based on the treatment plan - typically four to six implants per arch using concepts such as All-on-Four or All-on-Six [1,2]. If adequate primary stability (≥35 Ncm) is achieved, immediate provisionalization is carried out using a screw-retained acrylic prosthesis within 24-48 hours, allowing early function and improved patient comfort [6,7].

Following an osseointegration period of approximately three to four months, definitive prosthodontic steps are undertaken. These include accurate implant-level impressions, jaw relation records, a wax trial for esthetic and functional verification, and the delivery of the definitive prosthesis, which may be a hybrid metal-acrylic, monolithic zirconia, or metal-ceramic prosthesis. Occlusion is adjusted following the principles of implant-protected occlusion, ensuring light centric contacts, shallow anterior guidance, and minimal cantilever forces [14].

In cases where fixed prostheses are contraindicated or when patients prefer a removable option, implant-supported overdentures offer a reliable alternative. These prostheses typically utilize two to six implants and are retained using attachments such as locator abutments, bars with clips, ball attachments, or magnets, depending on the available interarch space, patient dexterity, and hygiene needs [1]. Implant-supported overdentures provide significantly improved retention, stability, and comfort compared to conventional complete dentures, while also offering ease of maintenance and affordability [16].

Both treatment modalities require meticulous planning, surgical precision, and prosthetic accuracy to ensure long-term success. Regular follow-up appointments are essential for monitoring peri-implant health, evaluating prosthesis function and integrity, and reinforcing oral hygiene practices [17].

Discussion

Full-mouth implant-supported rehabilitation has revolutionized the management of edentulous patients, offering enhanced function, esthetics, and quality of life [1]. The multitude of prosthetic options - from fixed full-arch restorations to implant-supported overdentures - provides clinicians the flexibility to tailor treatment plans based on patient-specific anatomical, functional, and economic considerations [1]. However, the choice of prosthetic design must be grounded in a comprehensive understanding of biomechanics, material science, patient expectations, and long-term outcomes [8,9,16].

Fixed vs Removable Prostheses: A Clinical Dilemma

The decision between fixed and removable prosthetic modalities remains a central aspect of full-arch rehabilitation planning. Fixed prostheses, especially All-on-Four and All-on-Six concepts, have demonstrated excellent survival rates, often exceeding 95% over 5-10 years [1,2]. These solutions are ideal for patients demanding a natural-feeling, immobile prosthesis and are compliant with long-term maintenance. However, they require higher initial investment and sufficient bone volume, especially in the posterior maxilla, to ensure predictable implant placement and load distribution.

In contrast, implant-retained overdentures - though more affordable and easier to maintain - may be less stable in function and are often associated with increased maintenance needs, such as periodic relining and attachment replacement [3,5]. Their suitability for elderly or medically compromised patients makes them an essential part of the prosthetic spectrum.

Biomechanical and Functional Considerations

Biomechanical load distribution plays a pivotal role in long-term success. FEA studies consistently show that the All-on-Six design distributes occlusal forces more evenly than All-on-Four, reducing the cantilever effect and prosthetic complications [2]. The angulation of posterior implants in All-on-Four maximizes anteroposterior spread but may concentrate stress at the bone-implant interface or prosthetic junction if not carefully planned.

For removable options, the attachment system critically affects load transmission and prosthesis stability. Bar attachments, although superior in retention, create challenges in hygiene and require more restorative space. Locator and stud attachments offer easier hygiene and patient adaptability but may compromise long-term retention due to wear or fracture of plastic components [3].

Material Selection: Esthetics vs Durability

The evolution of restorative materials has significantly influenced prosthetic success. Monolithic zirconia has gained popularity for its superior esthetics, high flexural strength, and resistance to chipping [4]. Nevertheless, its rigidity can lead to stress concentration, and wear on opposing dentition is a concern. Metal-acrylic hybrid prostheses, although cost-effective and easier to repair, may suffer from fractures or esthetic degradation over time.

Emerging materials, such as PEEK and high-performance polymers, offer flexibility and shock absorption, but their long-term clinical evidence remains limited [8,18]. The selection must therefore balance mechanical strength, esthetic demands, ease of repair, and patient budget.

Digital Workflows: Precision and Efficiency

Digital technology has streamlined full-arch implant workflows. Intraoral scanning, guided implant surgery, and CAD/CAM prosthesis fabrication improve accuracy, reduce chair time, and enhance patient satisfaction [16]. Digital planning also enables preoperative visualization of implant trajectories, vital in cases with compromised bone. However, digital workflows may require steep learning curves and infrastructure investment, and their effectiveness can be limited in patients with severe ridge resorption or restricted mouth opening [16].

The hybridization of digital and conventional protocols - such as using analog impressions in complex soft tissue cases, followed by digital design - provides the best of both worlds and ensures predictability in challenging scenarios [16].

Long-Term Maintenance and Complications

Long-term prosthetic success depends heavily on preventive maintenance. Fixed restorations, while stable, may be difficult to clean if contours are bulky or implant positions are suboptimal. Peri-implantitis remains a risk, particularly in cement-retained restorations with excess cement [3,4]. Screw-retained options allow retrievability and facilitate hygiene but may present with screw loosening or framework fractures [4].

Removable overdentures require frequent recall visits due to wear of attachment components and possible mucosal irritation. Patient education and adherence to hygiene protocols are crucial for minimizing biologic and mechanical complications.

Patient-Centered Treatment Planning

Ultimately, prosthetic decision-making must align with patient expectations, esthetic demands, anatomical feasibility, and financial capacity. Some patients may prioritize fixed esthetics and function at a higher cost, while others may prefer removable options for ease of hygiene and affordability. The use of digital smile design, mock-ups, and shared decision-making models improves treatment acceptance and outcome satisfaction [9,14,19].

In patients with minimal bone, resorbed ridges, or sinus pneumatization, zygomatic implants or staged grafting protocols may be needed to facilitate fixed prosthesis options [16]. Alternatively, high-performance removable prostheses - crafted from advanced materials such as PEEK or reinforced acrylics - offer a practical solution with minimal surgical morbidity, particularly in elderly or systemically compromised patients [18,19].

## Conclusions

Full-mouth implant rehabilitation provides tailored prosthetic solutions to meet each patient’s functional and esthetic needs. Fixed options such as All-on-Four frameworks and monolithic zirconia bridges offer durable, lifelike results, while removable overdentures remain cost-effective and hygienic choices, especially for elderly or medically compromised patients. Successful outcomes rely on careful planning - selecting suitable materials, attachment systems, and digital-analog workflows - supported by consistent maintenance. Digital integration enhances precision, efficiency, and patient satisfaction.

Global guidelines, including the McGill Consensus, endorse mandibular two-implant overdentures as the minimum standard for edentulous care. Advanced bone loss cases may require zygomatic implants or grafting to achieve fixed solutions. Ultimately, patient-centered planning grounded in sound biomechanics ensures long-term functional, esthetic, and psychological success.
